# 6-Bromo-4-(2-cyclo­hexyl­idenehydrazin-1-yl­idene)-1-methyl-2,2-dioxo-3,4-dihydro-1*H*-2λ^6^,1-benzothia­zine

**DOI:** 10.1107/S1600536812025123

**Published:** 2012-06-13

**Authors:** Muhammad Shafiq, Islam Ullah Khan, Muhammad Zia-ur-Rehman, Muhammad Nadeem Arshad, Muhammad Safder, Zeeshan Haider

**Affiliations:** a Department of Chemistry, Government College University, Faisalabad 38040, Pakistan; b Materials Chemistry Laboratory, Department of Chemistry, GC University, Lahore 54000, Pakistan; c Applied Chemistry Research Centre, PCSIR Laboratories Complex, Ferozpure Road, Lahore 54600, Pakistan; d Department of Chemistry, University of Gujrat, Gujrat 50781, Pakistan

## Abstract

The asymmetric unit of the title compound, C_15_H_18_BrN_3_O_2_S, contains two independent mol­ecules in both of which the (thia­zine)C=N—N double bond exhibits an *E* conformation. The cyclo­hexyl rings adopt chair conformations while the thia­zine rings are in sofa conformations. The mean planes of these rings are oriented at dihedral angles of 64.43 (13) and 28.6 (2)° in the two independent mol­ecules while the aromatic and thia­zine rings are twisted by dihedral angles of 8.73 (8) and 13.07 (2)°, respectively. In the crystal, C—H⋯O and C—H⋯Br inter­actions connect mol­ecules into chains propagating along the *a* axis.

## Related literature

For the synthesis of benzothia­zines and their derivatives, see: Arshad *et al.* (2010 ▶); Shafiq *et al.* (2011*a* ▶,*b* ▶). For their biological activity, see: Zia-ur-Rehman *et al.* (2009) ▶. For related structures, see: Shafiq *et al.* (2011*c* ▶,*d* ▶). For puckering parameters, see: Cremer & Pople (1975 ▶).
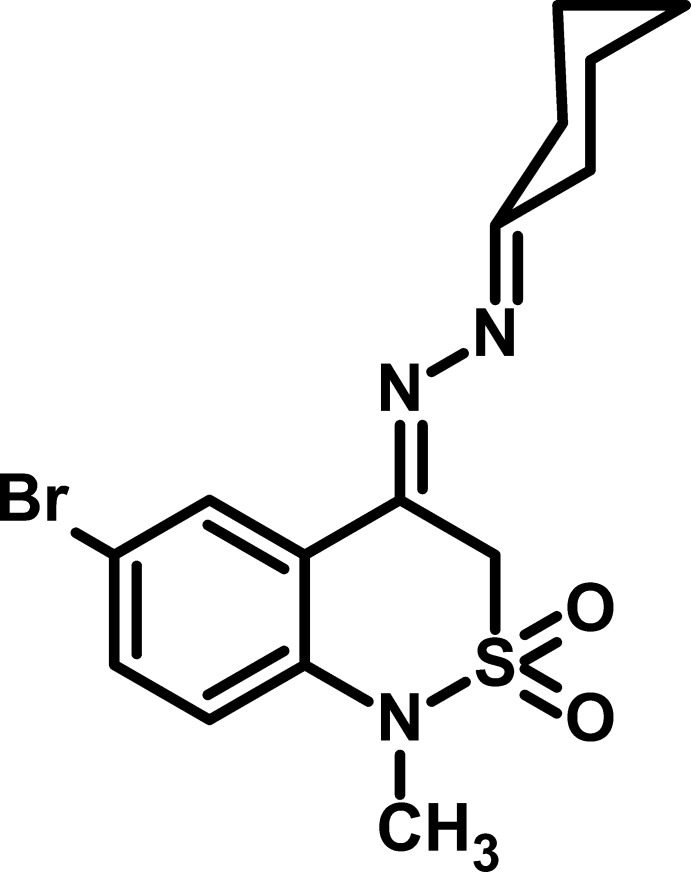


## Experimental

### 

#### Crystal data

C_15_H_18_BrN_3_O_2_S
*M* *_r_* = 384.29
Triclinic, 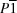

*a* = 9.9357 (2) Å
*b* = 11.2614 (3) Å
*c* = 15.8263 (3) Å
α = 110.625 (1)°
β = 91.525 (3)°
γ = 102.879 (4)°
*V* = 1604.85 (7) Å^3^
*Z* = 4
Mo *K*α radiation
μ = 2.70 mm^−1^
*T* = 296 K
0.25 × 0.21 × 0.13 mm

#### Data collection

Bruker KAPPA APEXII CCD diffractometer
Absorption correction: multi-scan (*SADABS*; Bruker, 2007 ▶) *T*_min_ = 0.552, *T*_max_ = 0.720
28996 measured reflections
7939 independent reflections
4380 reflections with *I* > 2σ(*I*)
*R*_int_ = 0.083

#### Refinement

*R*[*F* ^2^ > 2σ(*F* ^2^)] = 0.049
*wR*(*F* ^2^) = 0.125
*S* = 0.90
7939 reflections
399 parameters
H-atom parameters constrained
Δρ_max_ = 0.64 e Å^−3^
Δρ_min_ = −0.82 e Å^−3^

### 

Data collection: *APEX2* (Bruker, 2007 ▶); cell refinement: *SAINT* (Bruker, 2007 ▶); data reduction: *SAINT*; program(s) used to solve structure: *SHELXS97* (Sheldrick, 2008 ▶); program(s) used to refine structure: *SHELXL97* (Sheldrick, 2008 ▶); molecular graphics: *PLATON* (Spek, 2009 ▶); software used to prepare material for publication: *WinGX* (Farrugia, 1999 ▶) and *PLATON*.

## Supplementary Material

Crystal structure: contains datablock(s) I, global. DOI: 10.1107/S1600536812025123/im2384sup1.cif

Structure factors: contains datablock(s) I. DOI: 10.1107/S1600536812025123/im2384Isup2.hkl

Supplementary material file. DOI: 10.1107/S1600536812025123/im2384Isup3.cml

Additional supplementary materials:  crystallographic information; 3D view; checkCIF report

## Figures and Tables

**Table 1:** Hydrogen-bond geometry (Å, °)

*D*—H⋯*A*	*D*—H	H⋯*A*	*D*⋯*A*	*D*—H⋯*A*
C25—H25*A*⋯Br2^i^	0.97	2.84	3.752 (4)	157
C8—H8*A*⋯Br1^ii^	0.97	3.21	4.081 (3)	151
C18—H18⋯O1^iii^	0.93	2.59	3.332 (4)	137

Symmetry codes: (i) 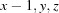; (ii) 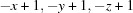; (iii) 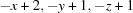.

**Fig. 1.**
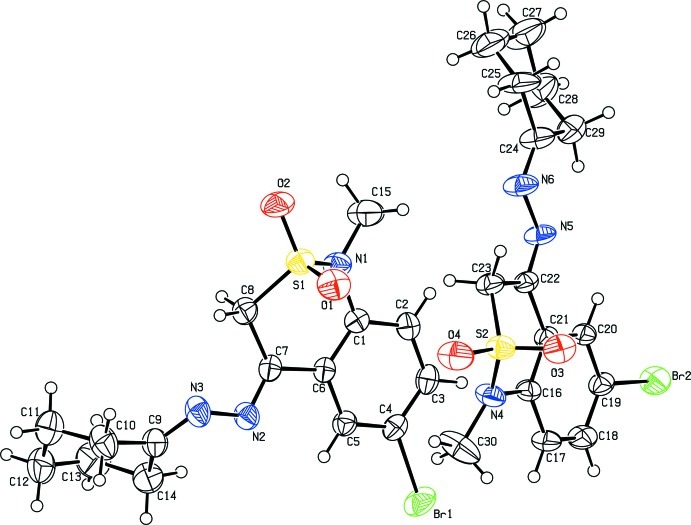
Molecular structure of (I) with thermal allipsoids drawn at the 40% probability level.

**Fig. 2.**
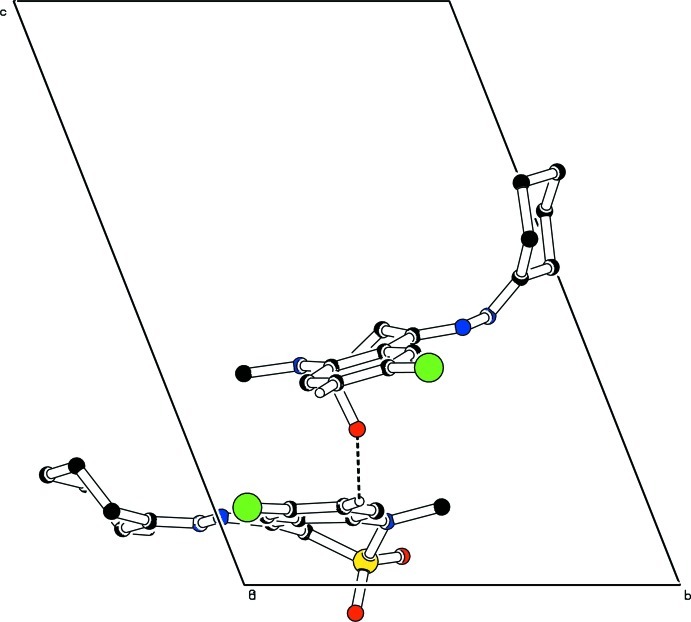
Perspective view showing the difference in dihedral angles between cyclohexyl rings with thiazine rings in both molecules.

**Fig. 3.**
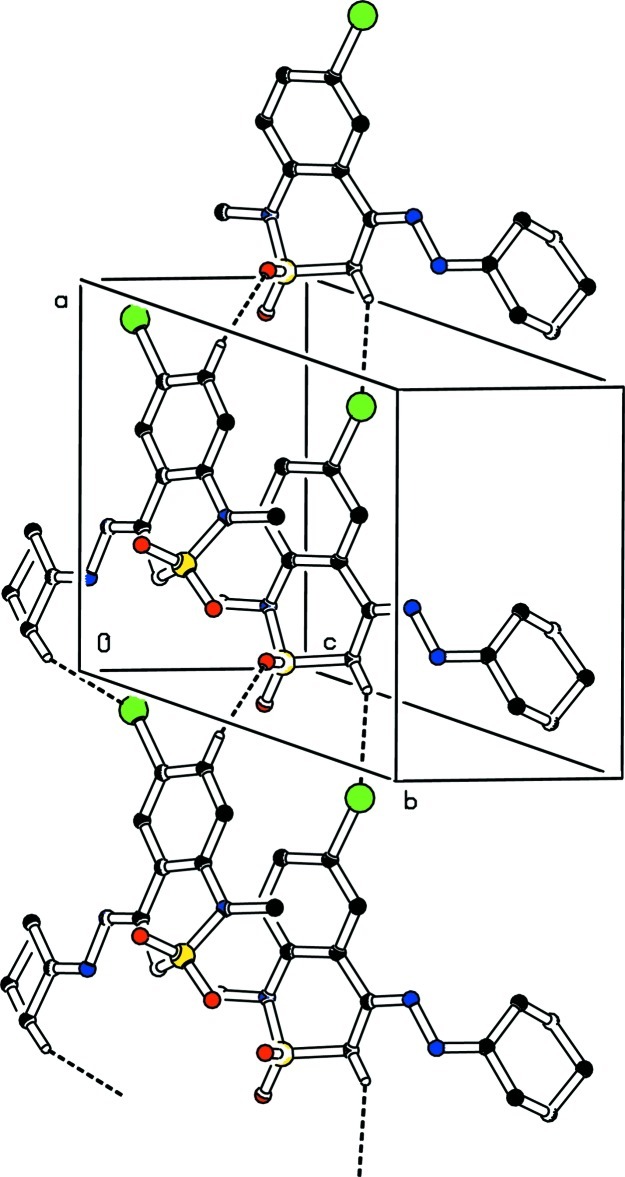
Unit cell packing showing weak interactions of hydrogen bonds using dashed lines.

## supplementary crystallographic information

### Comment

In perpetuation of our research regarding the synthesis of benzothiazines (Shafiq *et al.*, 2011*a*), (Arshad *et al.*, 2010), their derivatives (Shafiq *et al.*, 2011*b*) and biological evaluations (Zia-ur-Rehman *et al.*, 2009) we herein report the structural analysis of the title compound.

The present structure is closely related to 6-bromo-4-hydrazinylidene-1-methyl-3*H*-2λ^6^,1-benzothiazine-2,2-dione (Shafiq *et al.*, 2011*c*) and 6-bromo-1-methyl-4-[2- (4-methylbenzylidene) hydrazinylidene]-3*H*-2λ^6^,1-benzothiazine-2,2-dione (Shafiq *et al.*, 2011*d*). The crystal structure comprises of two independent molecules A (C1—C15) and B (C16—C30) per asymmetric unit. The cyclohexyl moieties adopt chair conformations with r. m. s. deviations of 0.228 (3)° and 0.223 (4)° while sofa conformations are observed for the thiazine rings with r. m. s. deviavtions of 0.235 (2)° and 0.236 (2)° in A and B, respectively (Fig. 1). The point of difference between the two molecules is the dihedral angles between the fused aromatic and thiazine rings which are 8.73 (8)° and 13.07 (2)°. Moreover, cyclohexyl rings are oriented at dihedral angles of 64.43 (13)° and 28.64 (20)° with respect to thiazine rings in molecules A and B, respectively (Fig. 2). Both thiazine rings show different total ring puckering amplitude values as QT = 0.576 Å with (θ) = 50.8 (3)° and (π) = 353.7 (4)° for molecule A and QT = 0.578 Å with (θ) = 122.6 (3)° and (π) = 186.2 (4)° for molecule B (Cremer & Pople, 1975). The molecules do not show any classical hydrogen bonding although weak intermolecular interactions of the C—H···O and C—H···Br type have been observed (Table. 1, Fig. 3).

### Experimental

In the synthesis of title compound, 6-Bromo-4-hydrazinylidene-1- methyl-3*H*-2λ^6^,1-benzothiazine-2,2-dione (Shafiq *et al.*, 2011*c*) was subjected to react with cyclohexanone according to a literature procedure (yield: 56.7%, Shafiq *et al.*, 2011*b*). The product obtained was then recrystallized from ethyl acetate by slow evaporation of the solvent to obtain suitable crystals for diffraction studies.

### Refinement

All hydrogen atoms were positioned with idealized geometry with C—H = 0.96 Å for the methyl group, C—H = 0.93 Å for aromatic and C—H = 0.97 Å for methylene groups and were refined using a riding model with *U*_iso_(H) = 1.2 *U*_eq_(C) for aromatic & methylene and *U*_iso_(H) = 1.5 *U*_eq_(C) for methyl carbon atoms. Six reflections (-1 0 1), (0 - 1 1), (0 1 0), (0 0 1), (1 0 0), (1 - 1 1) have been omitted in the final refinement as these were obscured by the beam stop.

### Crystal data

**Table:**

C_15_H_18_BrN_3_O_2_S	*Z* = 4
*M**_r_* = 384.29	*F*(000) = 784
Triclinic, *P*1	*D*_x_ = 1.591 Mg m^−^^3^
Hall symbol: -P 1	Mo *K*α radiation, λ = 0.71073 Å
*a* = 9.9357 (2) Å	Cell parameters from 8531 reflections
*b* = 11.2614 (3) Å	θ = 2.6–24.9°
*c* = 15.8263 (3) Å	µ = 2.70 mm^−^^1^
α = 110.625 (1)°	*T* = 296 K
β = 91.525 (3)°	Block, light yellow
γ = 102.879 (4)°	0.25 × 0.21 × 0.13 mm
*V* = 1604.85 (7) Å^3^	

### Data collection

**Table:**

Bruker KAPPA APEXII CCD diffractometer	7939 independent reflections
Radiation source: fine-focus sealed tube	4380 reflections with *I* > 2σ(*I*)
Graphite monochromator	*R*_int_ = 0.083
φ and ω scans	θ_max_ = 28.3°, θ_min_ = 2.5°
Absorption correction: multi-scan (*SADABS*; Bruker, 2007)	*h* = −13→13
*T*_min_ = 0.552, *T*_max_ = 0.720	*k* = −14→15
28996 measured reflections	*l* = −21→20

### Refinement

**Table:**

Refinement on *F*^2^	Primary atom site location: structure-invariant direct methods
Least-squares matrix: full	Secondary atom site location: difference Fourier map
*R*[*F*^2^ > 2σ(*F*^2^)] = 0.049	Hydrogen site location: inferred from neighbouring sites
*wR*(*F*^2^) = 0.125	H-atom parameters constrained
*S* = 0.90	*w* = 1/[σ^2^(*F*_o_^2^) + (0.0673*P*)^2^] where *P* = (*F*_o_^2^ + 2*F*_c_^2^)/3
7939 reflections	(Δ/σ)_max_ = 0.001
399 parameters	Δρ_max_ = 0.64 e Å^−^^3^
0 restraints	Δρ_min_ = −0.82 e Å^−^^3^

### Special details

**Table:**

Geometry. All e.s.d.'s (except the e.s.d. in the dihedral angle between two l.s. planes) are estimated using the full covariance matrix. The cell e.s.d.'s are taken into account individually in the estimation of e.s.d.'s in distances, angles and torsion angles; correlations between e.s.d.'s in cell parameters are only used when they are defined by crystal symmetry. An approximate (isotropic) treatment of cell e.s.d.'s is used for estimating e.s.d.'s involving l.s. planes.
Refinement. Refinement of *F*^2^ against ALL reflections. The weighted *R*-factor *wR* and goodness of fit *S* are based on *F*^2^, conventional *R*-factors *R* are based on *F*, with *F* set to zero for negative *F*^2^. The threshold expression of *F*^2^ > σ(*F*^2^) is used only for calculating *R*-factors(gt) *etc*. and is not relevant to the choice of reflections for refinement. *R*-factors based on *F*^2^ are statistically about twice as large as those based on *F*, and *R*- factors based on ALL data will be even larger.

### Fractional atomic coordinates and isotropic or equivalent isotropic displacement parameters (Å^2^)

**Table:**

	*x*	*y*	*z*	*U*_iso_*/*U*_eq_	
Br1	0.15281 (3)	0.37923 (4)	0.62774 (3)	0.06860 (15)	
Br2	0.91986 (3)	0.07710 (3)	0.13278 (3)	0.06029 (14)	
S1	0.87214 (8)	0.61054 (8)	0.64609 (6)	0.0502 (2)	
S2	0.35514 (8)	0.29988 (8)	0.04039 (6)	0.0484 (2)	
O1	0.8657 (2)	0.5997 (2)	0.73305 (15)	0.0632 (6)	
O2	0.9971 (2)	0.6817 (2)	0.62658 (19)	0.0695 (7)	
O3	0.3870 (2)	0.2295 (2)	−0.04815 (15)	0.0605 (6)	
O4	0.2674 (2)	0.3874 (2)	0.04923 (19)	0.0701 (7)	
N1	0.7451 (3)	0.6722 (3)	0.62437 (18)	0.0496 (7)	
N2	0.6362 (3)	0.2653 (3)	0.55860 (18)	0.0500 (7)	
N3	0.7411 (3)	0.1977 (3)	0.53974 (19)	0.0553 (7)	
N4	0.4992 (3)	0.3844 (2)	0.1089 (2)	0.0530 (7)	
N5	0.3694 (3)	0.0088 (3)	0.11621 (19)	0.0500 (7)	
N6	0.2248 (3)	−0.0462 (3)	0.1053 (2)	0.0602 (8)	
C1	0.6078 (3)	0.6025 (3)	0.62716 (19)	0.0390 (7)	
C2	0.5052 (3)	0.6705 (3)	0.6535 (2)	0.0463 (8)	
H2	0.5286	0.7612	0.6714	0.056*	
C3	0.3695 (3)	0.6060 (3)	0.6537 (2)	0.0479 (8)	
H3	0.3018	0.6522	0.6710	0.058*	
C4	0.3370 (3)	0.4723 (3)	0.6278 (2)	0.0438 (7)	
C5	0.4364 (3)	0.4031 (3)	0.6025 (2)	0.0424 (7)	
H5	0.4120	0.3126	0.5862	0.051*	
C6	0.5730 (3)	0.4666 (3)	0.60093 (18)	0.0365 (7)	
C7	0.6755 (3)	0.3872 (3)	0.57341 (18)	0.0401 (7)	
C8	0.8190 (3)	0.4518 (3)	0.5629 (2)	0.0483 (8)	
H8A	0.8209	0.4565	0.5029	0.058*	
H8B	0.8826	0.4005	0.5690	0.058*	
C9	0.7090 (3)	0.0879 (3)	0.4734 (2)	0.0500 (8)	
C10	0.8150 (4)	0.0082 (4)	0.4558 (3)	0.0683 (10)	
H10A	0.8998	0.0582	0.4962	0.082*	
H10B	0.7803	−0.0707	0.4685	0.082*	
C11	0.8463 (4)	−0.0284 (4)	0.3592 (3)	0.0821 (13)	
H11A	0.9064	−0.0882	0.3477	0.099*	
H11B	0.8957	0.0496	0.3496	0.099*	
C12	0.7159 (5)	−0.0924 (4)	0.2927 (3)	0.0837 (13)	
H12A	0.6692	−0.1738	0.2989	0.100*	
H12B	0.7399	−0.1124	0.2311	0.100*	
C13	0.6195 (5)	−0.0004 (4)	0.3113 (3)	0.0828 (12)	
H13A	0.6654	0.0797	0.3031	0.099*	
H13B	0.5360	−0.0414	0.2684	0.099*	
C14	0.5809 (4)	0.0316 (4)	0.4074 (3)	0.0654 (10)	
H14A	0.5237	0.0939	0.4196	0.078*	
H14B	0.5276	−0.0474	0.4140	0.078*	
C15	0.7765 (4)	0.8096 (4)	0.6379 (3)	0.0755 (12)	
H15A	0.7728	0.8600	0.7006	0.113*	
H15B	0.7097	0.8245	0.6004	0.113*	
H15C	0.8680	0.8356	0.6219	0.113*	
C16	0.5950 (3)	0.3106 (3)	0.1156 (2)	0.0401 (7)	
C17	0.7380 (3)	0.3686 (3)	0.1268 (2)	0.0498 (8)	
H17	0.7686	0.4541	0.1290	0.060*	
C18	0.8332 (3)	0.3004 (3)	0.1347 (2)	0.0500 (8)	
H18	0.9280	0.3397	0.1434	0.060*	
C19	0.7868 (3)	0.1732 (3)	0.1296 (2)	0.0430 (7)	
C20	0.6479 (3)	0.1149 (3)	0.11944 (18)	0.0388 (7)	
H20	0.6187	0.0288	0.1161	0.047*	
C21	0.5492 (3)	0.1845 (3)	0.11393 (18)	0.0367 (7)	
C22	0.4002 (3)	0.1204 (3)	0.10748 (19)	0.0382 (7)	
C23	0.2919 (3)	0.1877 (3)	0.0914 (2)	0.0475 (8)	
H23A	0.2612	0.2328	0.1490	0.057*	
H23B	0.2121	0.1223	0.0529	0.057*	
C24	0.1940 (3)	−0.1601 (4)	0.1095 (3)	0.0572 (9)	
C25	0.0414 (4)	−0.2278 (4)	0.0967 (3)	0.0832 (14)	
H25A	−0.0134	−0.1680	0.0931	0.100*	
H25B	0.0201	−0.3028	0.0399	0.100*	
C26	0.0037 (5)	−0.2716 (5)	0.1723 (4)	0.1089 (18)	
H26A	−0.0917	−0.3237	0.1587	0.131*	
H26B	0.0096	−0.1956	0.2272	0.131*	
C27	0.0976 (5)	−0.3516 (5)	0.1886 (4)	0.1043 (17)	
H27A	0.0738	−0.3734	0.2413	0.125*	
H27B	0.0838	−0.4328	0.1365	0.125*	
C28	0.2471 (4)	−0.2771 (5)	0.2039 (3)	0.0858 (13)	
H28A	0.2620	−0.1995	0.2588	0.103*	
H28B	0.3056	−0.3312	0.2126	0.103*	
C29	0.2878 (4)	−0.2377 (4)	0.1263 (3)	0.0639 (10)	
H29A	0.2817	−0.3150	0.0723	0.077*	
H29B	0.3831	−0.1856	0.1397	0.077*	
C30	0.5410 (5)	0.5235 (3)	0.1337 (3)	0.0853 (14)	
H30A	0.5847	0.5634	0.1952	0.128*	
H30B	0.4609	0.5560	0.1286	0.128*	
H30C	0.6055	0.5445	0.0939	0.128*	

### Atomic displacement parameters (Å^2^)

**Table:**

	*U*^11^	*U*^22^	*U*^33^	*U*^12^	*U*^13^	*U*^23^
Br1	0.0334 (2)	0.0842 (3)	0.0951 (3)	0.01748 (19)	0.01696 (18)	0.0388 (2)
Br2	0.03317 (19)	0.0630 (2)	0.0787 (3)	0.01020 (16)	−0.00475 (16)	0.02066 (19)
S1	0.0331 (4)	0.0531 (5)	0.0562 (5)	0.0042 (4)	−0.0052 (4)	0.0148 (4)
S2	0.0372 (4)	0.0466 (5)	0.0631 (5)	0.0076 (4)	−0.0008 (4)	0.0244 (4)
O1	0.0537 (15)	0.0742 (16)	0.0523 (14)	0.0061 (13)	−0.0159 (11)	0.0196 (12)
O2	0.0376 (14)	0.0679 (16)	0.095 (2)	−0.0009 (12)	0.0068 (13)	0.0282 (14)
O3	0.0568 (15)	0.0702 (15)	0.0562 (14)	0.0085 (12)	−0.0007 (11)	0.0302 (12)
O4	0.0488 (15)	0.0611 (15)	0.109 (2)	0.0164 (12)	−0.0021 (14)	0.0413 (15)
N1	0.0383 (15)	0.0487 (16)	0.0631 (17)	0.0087 (13)	0.0031 (13)	0.0237 (13)
N2	0.0390 (15)	0.0467 (16)	0.0614 (17)	0.0168 (13)	0.0036 (13)	0.0128 (13)
N3	0.0438 (16)	0.0534 (17)	0.0643 (18)	0.0243 (14)	0.0001 (13)	0.0093 (15)
N4	0.0394 (16)	0.0354 (15)	0.0760 (19)	0.0021 (12)	−0.0052 (14)	0.0161 (13)
N5	0.0254 (13)	0.0530 (17)	0.0722 (19)	−0.0032 (12)	0.0004 (12)	0.0320 (15)
N6	0.0285 (15)	0.0593 (19)	0.098 (2)	−0.0002 (13)	0.0013 (14)	0.0416 (18)
C1	0.0361 (17)	0.0449 (18)	0.0365 (16)	0.0088 (14)	−0.0001 (12)	0.0166 (13)
C2	0.050 (2)	0.0460 (18)	0.0438 (17)	0.0181 (16)	0.0061 (15)	0.0143 (14)
C3	0.0431 (19)	0.059 (2)	0.0473 (18)	0.0260 (17)	0.0089 (14)	0.0182 (16)
C4	0.0361 (17)	0.057 (2)	0.0405 (16)	0.0123 (15)	0.0063 (13)	0.0201 (15)
C5	0.0359 (17)	0.0468 (18)	0.0452 (17)	0.0126 (14)	0.0050 (13)	0.0163 (14)
C6	0.0322 (16)	0.0442 (17)	0.0320 (14)	0.0127 (13)	0.0006 (12)	0.0111 (13)
C7	0.0315 (16)	0.051 (2)	0.0338 (15)	0.0125 (14)	−0.0008 (12)	0.0095 (14)
C8	0.0338 (17)	0.0529 (19)	0.0552 (19)	0.0145 (15)	0.0023 (14)	0.0143 (16)
C9	0.050 (2)	0.053 (2)	0.0495 (19)	0.0191 (17)	0.0053 (15)	0.0184 (17)
C10	0.067 (3)	0.062 (2)	0.077 (3)	0.034 (2)	0.002 (2)	0.0162 (19)
C11	0.072 (3)	0.079 (3)	0.096 (3)	0.031 (2)	0.028 (3)	0.023 (3)
C12	0.110 (4)	0.079 (3)	0.054 (2)	0.024 (3)	0.027 (2)	0.013 (2)
C13	0.081 (3)	0.093 (3)	0.060 (3)	0.013 (3)	−0.009 (2)	0.017 (2)
C14	0.049 (2)	0.069 (2)	0.071 (2)	0.0138 (19)	0.0004 (18)	0.0177 (19)
C15	0.058 (2)	0.063 (3)	0.112 (3)	0.008 (2)	0.010 (2)	0.044 (2)
C16	0.0337 (16)	0.0363 (17)	0.0435 (17)	0.0012 (14)	−0.0001 (13)	0.0114 (13)
C17	0.0394 (18)	0.0411 (18)	0.061 (2)	−0.0049 (15)	−0.0010 (15)	0.0181 (16)
C18	0.0311 (17)	0.054 (2)	0.054 (2)	−0.0029 (15)	−0.0071 (14)	0.0152 (16)
C19	0.0295 (16)	0.0491 (19)	0.0459 (17)	0.0037 (14)	0.0015 (13)	0.0160 (14)
C20	0.0325 (16)	0.0373 (16)	0.0390 (16)	0.0012 (13)	0.0010 (12)	0.0098 (13)
C21	0.0257 (15)	0.0414 (17)	0.0351 (15)	−0.0005 (13)	0.0011 (12)	0.0100 (13)
C22	0.0294 (15)	0.0422 (18)	0.0399 (16)	0.0022 (13)	0.0032 (12)	0.0156 (13)
C23	0.0316 (17)	0.0493 (19)	0.062 (2)	0.0033 (14)	0.0055 (15)	0.0249 (16)
C24	0.0360 (19)	0.061 (2)	0.080 (2)	−0.0020 (17)	0.0003 (17)	0.042 (2)
C25	0.038 (2)	0.074 (3)	0.145 (4)	−0.0092 (19)	0.003 (2)	0.063 (3)
C26	0.067 (3)	0.098 (4)	0.181 (5)	0.020 (3)	0.065 (3)	0.071 (4)
C27	0.083 (3)	0.110 (4)	0.165 (5)	0.033 (3)	0.058 (3)	0.097 (4)
C28	0.075 (3)	0.109 (3)	0.109 (4)	0.037 (3)	0.030 (3)	0.073 (3)
C29	0.048 (2)	0.070 (2)	0.074 (2)	0.0146 (19)	0.0162 (18)	0.027 (2)
C30	0.091 (3)	0.041 (2)	0.110 (3)	0.010 (2)	−0.036 (3)	0.018 (2)

### Geometric parameters (Å, º)

**Table:**

Br1—C4	1.895 (3)	C12—H12A	0.9700
Br2—C19	1.896 (3)	C12—H12B	0.9700
S1—O2	1.425 (2)	C13—C14	1.516 (5)
S1—O1	1.426 (2)	C13—H13A	0.9700
S1—N1	1.652 (3)	C13—H13B	0.9700
S1—C8	1.758 (3)	C14—H14A	0.9700
S2—O4	1.429 (2)	C14—H14B	0.9700
S2—O3	1.430 (2)	C15—H15A	0.9600
S2—N4	1.647 (3)	C15—H15B	0.9600
S2—C23	1.742 (3)	C15—H15C	0.9600
N1—C1	1.425 (4)	C16—C21	1.382 (4)
N1—C15	1.444 (4)	C16—C17	1.403 (4)
N2—C7	1.273 (4)	C17—C18	1.374 (4)
N2—N3	1.403 (3)	C17—H17	0.9300
N3—C9	1.275 (4)	C18—C19	1.376 (4)
N4—C16	1.420 (4)	C18—H18	0.9300
N4—C30	1.432 (4)	C19—C20	1.368 (4)
N5—C22	1.283 (4)	C20—C21	1.404 (4)
N5—N6	1.411 (3)	C20—H20	0.9300
N6—C24	1.276 (4)	C21—C22	1.479 (4)
C1—C6	1.394 (4)	C22—C23	1.510 (4)
C1—C2	1.395 (4)	C23—H23A	0.9700
C2—C3	1.382 (4)	C23—H23B	0.9700
C2—H2	0.9300	C24—C29	1.489 (5)
C3—C4	1.371 (4)	C24—C25	1.510 (5)
C3—H3	0.9300	C25—C26	1.474 (6)
C4—C5	1.374 (4)	C25—H25A	0.9700
C5—C6	1.392 (4)	C25—H25B	0.9700
C5—H5	0.9300	C26—C27	1.509 (6)
C6—C7	1.477 (4)	C26—H26A	0.9700
C7—C8	1.491 (4)	C26—H26B	0.9700
C8—H8A	0.9700	C27—C28	1.500 (6)
C8—H8B	0.9700	C27—H27A	0.9700
C9—C14	1.488 (5)	C27—H27B	0.9700
C9—C10	1.502 (4)	C28—C29	1.483 (5)
C10—C11	1.498 (5)	C28—H28A	0.9700
C10—H10A	0.9700	C28—H28B	0.9700
C10—H10B	0.9700	C29—H29A	0.9700
C11—C12	1.510 (6)	C29—H29B	0.9700
C11—H11A	0.9700	C30—H30A	0.9600
C11—H11B	0.9700	C30—H30B	0.9600
C12—C13	1.523 (6)	C30—H30C	0.9600
			
O2—S1—O1	119.64 (16)	C9—C14—H14A	109.7
O2—S1—N1	107.23 (15)	C13—C14—H14A	109.7
O1—S1—N1	110.06 (14)	C9—C14—H14B	109.7
O2—S1—C8	110.50 (16)	C13—C14—H14B	109.7
O1—S1—C8	107.92 (15)	H14A—C14—H14B	108.2
N1—S1—C8	99.65 (15)	N1—C15—H15A	109.5
O4—S2—O3	118.78 (15)	N1—C15—H15B	109.5
O4—S2—N4	107.06 (15)	H15A—C15—H15B	109.5
O3—S2—N4	110.31 (14)	N1—C15—H15C	109.5
O4—S2—C23	110.93 (15)	H15A—C15—H15C	109.5
O3—S2—C23	108.25 (15)	H15B—C15—H15C	109.5
N4—S2—C23	99.86 (15)	C21—C16—C17	119.9 (3)
C1—N1—C15	121.1 (3)	C21—C16—N4	120.9 (3)
C1—N1—S1	116.1 (2)	C17—C16—N4	119.2 (3)
C15—N1—S1	118.5 (2)	C18—C17—C16	120.6 (3)
C7—N2—N3	115.1 (3)	C18—C17—H17	119.7
C9—N3—N2	115.5 (3)	C16—C17—H17	119.7
C16—N4—C30	121.9 (3)	C17—C18—C19	119.2 (3)
C16—N4—S2	115.3 (2)	C17—C18—H18	120.4
C30—N4—S2	119.6 (2)	C19—C18—H18	120.4
C22—N5—N6	113.0 (3)	C20—C19—C18	121.3 (3)
C24—N6—N5	113.2 (3)	C20—C19—Br2	120.2 (2)
C6—C1—C2	119.3 (3)	C18—C19—Br2	118.5 (2)
C6—C1—N1	121.0 (3)	C19—C20—C21	120.3 (3)
C2—C1—N1	119.6 (3)	C19—C20—H20	119.9
C3—C2—C1	121.4 (3)	C21—C20—H20	119.9
C3—C2—H2	119.3	C16—C21—C20	118.7 (3)
C1—C2—H2	119.3	C16—C21—C22	122.6 (3)
C4—C3—C2	118.6 (3)	C20—C21—C22	118.7 (3)
C4—C3—H3	120.7	N5—C22—C21	117.5 (3)
C2—C3—H3	120.7	N5—C22—C23	123.1 (3)
C3—C4—C5	121.2 (3)	C21—C22—C23	119.5 (3)
C3—C4—Br1	120.3 (2)	C22—C23—S2	112.2 (2)
C5—C4—Br1	118.6 (2)	C22—C23—H23A	109.2
C4—C5—C6	120.9 (3)	S2—C23—H23A	109.2
C4—C5—H5	119.6	C22—C23—H23B	109.2
C6—C5—H5	119.6	S2—C23—H23B	109.2
C5—C6—C1	118.6 (3)	H23A—C23—H23B	107.9
C5—C6—C7	118.5 (3)	N6—C24—C29	129.2 (3)
C1—C6—C7	122.9 (3)	N6—C24—C25	116.9 (3)
N2—C7—C6	117.9 (3)	C29—C24—C25	114.0 (3)
N2—C7—C8	122.9 (3)	C26—C25—C24	111.2 (4)
C6—C7—C8	119.2 (3)	C26—C25—H25A	109.4
C7—C8—S1	110.1 (2)	C24—C25—H25A	109.4
C7—C8—H8A	109.6	C26—C25—H25B	109.4
S1—C8—H8A	109.6	C24—C25—H25B	109.4
C7—C8—H8B	109.6	H25A—C25—H25B	108.0
S1—C8—H8B	109.6	C25—C26—C27	111.9 (3)
H8A—C8—H8B	108.2	C25—C26—H26A	109.2
N3—C9—C14	127.6 (3)	C27—C26—H26A	109.2
N3—C9—C10	116.9 (3)	C25—C26—H26B	109.2
C14—C9—C10	115.4 (3)	C27—C26—H26B	109.2
C11—C10—C9	110.9 (3)	H26A—C26—H26B	107.9
C11—C10—H10A	109.5	C28—C27—C26	110.7 (4)
C9—C10—H10A	109.5	C28—C27—H27A	109.5
C11—C10—H10B	109.5	C26—C27—H27A	109.5
C9—C10—H10B	109.5	C28—C27—H27B	109.5
H10A—C10—H10B	108.0	C26—C27—H27B	109.5
C10—C11—C12	112.1 (3)	H27A—C27—H27B	108.1
C10—C11—H11A	109.2	C29—C28—C27	111.8 (4)
C12—C11—H11A	109.2	C29—C28—H28A	109.3
C10—C11—H11B	109.2	C27—C28—H28A	109.3
C12—C11—H11B	109.2	C29—C28—H28B	109.3
H11A—C11—H11B	107.9	C27—C28—H28B	109.3
C11—C12—C13	109.6 (3)	H28A—C28—H28B	107.9
C11—C12—H12A	109.7	C28—C29—C24	109.7 (3)
C13—C12—H12A	109.7	C28—C29—H29A	109.7
C11—C12—H12B	109.7	C24—C29—H29A	109.7
C13—C12—H12B	109.7	C28—C29—H29B	109.7
H12A—C12—H12B	108.2	C24—C29—H29B	109.7
C14—C13—C12	110.3 (3)	H29A—C29—H29B	108.2
C14—C13—H13A	109.6	N4—C30—H30A	109.5
C12—C13—H13A	109.6	N4—C30—H30B	109.5
C14—C13—H13B	109.6	H30A—C30—H30B	109.5
C12—C13—H13B	109.6	N4—C30—H30C	109.5
H13A—C13—H13B	108.1	H30A—C30—H30C	109.5
C9—C14—C13	109.9 (3)	H30B—C30—H30C	109.5
			
O2—S1—N1—C1	171.4 (2)	C14—C9—C10—C11	50.5 (5)
O1—S1—N1—C1	−57.0 (3)	C9—C10—C11—C12	−52.2 (5)
C8—S1—N1—C1	56.3 (2)	C10—C11—C12—C13	57.8 (5)
O2—S1—N1—C15	−31.5 (3)	C11—C12—C13—C14	−59.5 (5)
O1—S1—N1—C15	100.1 (3)	N3—C9—C14—C13	123.9 (4)
C8—S1—N1—C15	−146.7 (3)	C10—C9—C14—C13	−52.7 (4)
C7—N2—N3—C9	−135.3 (3)	C12—C13—C14—C9	56.2 (5)
O4—S2—N4—C16	174.6 (2)	C30—N4—C16—C21	163.4 (3)
O3—S2—N4—C16	−54.8 (3)	S2—N4—C16—C21	−36.6 (4)
C23—S2—N4—C16	59.0 (3)	C30—N4—C16—C17	−14.6 (5)
O4—S2—N4—C30	−24.9 (4)	S2—N4—C16—C17	145.4 (3)
O3—S2—N4—C30	105.7 (3)	C21—C16—C17—C18	1.1 (5)
C23—S2—N4—C30	−140.5 (3)	N4—C16—C17—C18	179.2 (3)
C22—N5—N6—C24	176.7 (3)	C16—C17—C18—C19	1.3 (5)
C15—N1—C1—C6	170.4 (3)	C17—C18—C19—C20	−1.8 (5)
S1—N1—C1—C6	−33.2 (4)	C17—C18—C19—Br2	176.0 (2)
C15—N1—C1—C2	−7.4 (4)	C18—C19—C20—C21	0.0 (4)
S1—N1—C1—C2	149.0 (2)	Br2—C19—C20—C21	−177.8 (2)
C6—C1—C2—C3	−0.4 (4)	C17—C16—C21—C20	−2.9 (4)
N1—C1—C2—C3	177.4 (3)	N4—C16—C21—C20	179.1 (3)
C1—C2—C3—C4	0.5 (4)	C17—C16—C21—C22	176.5 (3)
C2—C3—C4—C5	0.2 (4)	N4—C16—C21—C22	−1.5 (4)
C2—C3—C4—Br1	179.5 (2)	C19—C20—C21—C16	2.4 (4)
C3—C4—C5—C6	−1.0 (5)	C19—C20—C21—C22	−177.0 (2)
Br1—C4—C5—C6	179.7 (2)	N6—N5—C22—C21	−177.6 (2)
C4—C5—C6—C1	1.1 (4)	N6—N5—C22—C23	2.6 (4)
C4—C5—C6—C7	179.8 (3)	C16—C21—C22—N5	−172.7 (3)
C2—C1—C6—C5	−0.4 (4)	C20—C21—C22—N5	6.7 (4)
N1—C1—C6—C5	−178.2 (3)	C16—C21—C22—C23	7.1 (4)
C2—C1—C6—C7	−179.0 (3)	C20—C21—C22—C23	−173.5 (3)
N1—C1—C6—C7	3.1 (4)	N5—C22—C23—S2	−157.0 (3)
N3—N2—C7—C6	−175.2 (2)	C21—C22—C23—S2	23.1 (3)
N3—N2—C7—C8	5.6 (4)	O4—S2—C23—C22	−163.5 (2)
C5—C6—C7—N2	−4.9 (4)	O3—S2—C23—C22	64.5 (3)
C1—C6—C7—N2	173.8 (3)	N4—S2—C23—C22	−50.9 (3)
C5—C6—C7—C8	174.3 (3)	N5—N6—C24—C29	1.8 (5)
C1—C6—C7—C8	−7.0 (4)	N5—N6—C24—C25	−178.8 (3)
N2—C7—C8—S1	−144.3 (3)	N6—C24—C25—C26	−127.0 (4)
C6—C7—C8—S1	36.6 (3)	C29—C24—C25—C26	52.5 (5)
O2—S1—C8—C7	−169.1 (2)	C24—C25—C26—C27	−52.1 (6)
O1—S1—C8—C7	58.4 (3)	C25—C26—C27—C28	55.2 (6)
N1—S1—C8—C7	−56.5 (2)	C26—C27—C28—C29	−57.4 (6)
N2—N3—C9—C14	8.1 (5)	C27—C28—C29—C24	56.2 (5)
N2—N3—C9—C10	−175.5 (3)	N6—C24—C29—C28	125.5 (4)
N3—C9—C10—C11	−126.4 (4)	C25—C24—C29—C28	−53.9 (5)

### Hydrogen-bond geometry (Å, º)

**Table:**

*D*—H···*A*	*D*—H	H···*A*	*D*···*A*	*D*—H···*A*
C25—H25*A*···Br2^i^	0.97	2.84	3.752 (4)	157
C8—H8*A*···Br1^ii^	0.97	3.21	4.081 (3)	151
C18—H18···O1^iii^	0.93	2.59	3.332 (4)	137

Symmetry codes: (i) *x*−1, *y*, *z*; (ii) −*x*+1, −*y*+1, −*z*+1; (iii) −*x*+2, −*y*+1, −*z*+1.
